# Domestic violence against Iranian pregnant adolescents: protective and risk factors

**DOI:** 10.4314/ahs.v23i4.43

**Published:** 2023-12

**Authors:** Mona Rahnavardi, Zahra Bostani Khalesi, Zahra hazrati

**Affiliations:** 1 Social Determinants of Health Research Center, Guilan University of Medical Sciences, Rasht, Iran; 2 Shahid Nourani Hospital, Guilan University of Medical Sciences, Rasht, Iran

**Keywords:** Domestic violence pregnant women, risk factors, adolescent

## Abstract

**Background:**

Domestic violence is an important health, which has serious impacts on women's health. The study aimed to discover the protective and risk factors of domestic violence among pregnant adolescents.

**Materials and Methods:**

In the analytical cross-sectional study, 255 eligible pregnant women aged 14–20 years who have been referred to Al-Zahra Hospital, in northern Iran between September 2020 and March 2022 participated. Demographical characteristics were recorded by a questionnaire. Domestic violence was assessed using the instrument of violence against women. Descriptive statistics were used to describe the basic features of the data.

**Results:**

The frequency of physical, emotional, and sexual violence was respectively 13.33%, 23.52%, and 9.01%. The risk factors included the spouse's substance use (OR 2.41, 95% CI 1.25–4.62), spouse's low education (OR 1.41, 95% CI 1.12–3.52), spouse's unemployment (OR 1.14, 95% CI 1.03–1.57) and domestic violence exposure in childhood (OR 1.85, 95% CI 1.46–2.51). Higher education for women was a protective factor for domestic violence (OR 0.70, 95% CI 0.45–0.83).

**Conclusions:**

The education level is a protective factor against domestic violence among pregnant adolescents. These results can help to design the most appropriate prevention programs to reduce the risk factors for violence among pregnant adolescents.

## Introduction

Domestic violence is a public health problem that can lead to harmful health effects and highly pervasive social problems, 1 and a violation of human rights.^2^ Available studies suggest that violence has linkages with age, 3 indeed, adults were less likely to be victims of violence than adolescents.^4^ After Barter et al., report on ‘Partner exploitation and violence in teenage intimate relationships’ considerable attention was paid to the issue of violence and abuse against adolescents.^5^ This is because violence is thought to have devastating effects on adolescents that can continue into adulthood.^6^ Stöckl et al., 7 reported that female adolescents were more likely to incur injuries related to domestic violence than were other age groups.

Adolescence and early adulthood are critical times for experiencing violence.^8^ Adolescence is a transitional stage that is associated with significant psychological and physical changes.^6^,^9^ It is the time in a youth's life when, in addition to coming to grips with the confusion and challenges of a maturing, he/she seeks answers to the many questions regarding identity versus role confusion, according to the psychosocial stages of development.^10^ Domestic violence can have serious and long-lasting effects on the mental health of adolescents. 11, 12 Smith et al., 13 reported that 43.2% of women first experienced a form of violence before age of 18 years. Furthermore, domestic violence is grounded in normative gender hierarchies, patriarchal systems of power, and ideologies of gender inequality.^14^ Special attention to gender issues is fundamental to the research of violence.^15^ A number of studies around the world have demonstrated that female than male adolescents are more likely to be exposed to abuse and violence.^9^, ^11^, ^16^ Many female adolescents who were victims of domestic violence suffer from prolonged periods of fear, anxiety disorders, severe depression, and intrusive thoughts related to the abuse.^6^ The effects of abuse and violence on pregnant adolescents are more severe and typically have been associated with fetal injury, miscarriage, preterm labor, and stillbirth. 17, 18 Because of adolescent pregnancies are potentially associated with serious social and health challenges such as isolation, poverty, low education, early divorce, and pregnancy outcomes.^19^ On the other, pregnant teenagers who were experienced physically assaulted were more likely than other women to “report that they had been injured, received medical treatment, received mental health counseling, lost time from work, and sought justice-system interventions as a result of their most recent victimization.^20^

While previous studies have shown some relationship between domestic violence victimization and individuals' low age, the protective and risk factors of domestic violence are yet to be explored in pregnant adolescents and we have a limited understanding of the protective and risk factors of violence against pregnant teenagers, and victimization and identify the causal mechanisms linking age with outcomes in female adolescents where substantial gaps and insufficient information in the research literature exist. For these reasons, the study aimed to discover the protective and risk factors of domestic violence among pregnant adolescents. Based on the research objective, the research question is raised, what factors predict domestic violence among pregnant adolescents?

## Methods

In the analytical cross-sectional study, 255 eligible pregnant women aged 14-20 years who have been referred to Al-Zahra Hospital, in northern Iran between September 2020 and March 2022 participated. A ratio estimation formula was used for the sample size calculation in the current study. The required sample size is obtained using the prevalence of domestic violence among pregnant women (p = 0.64, α = 0.05, and d = 10 %) reported by Tavoli et al. 21 Assuming a non-response rate of 10%, the final adjusted sample size was 258.

Participation in the study was voluntary and Research participants were informed that the information they provided would be kept confidential and anonymous. The participants were informed of their rights and roles in the study beforehand. A copy of the participant information sheet and consent form was given to participants. Women who agreed to participate in the study were directed to the researchers who were collecting the data. Criteria for selecting women to participate included the willingness to participate based on informed consent, ability to speak the Persian language, does not have an incurable disease (cancer and AIDS), does not abuse drugs (narcotics… smoking, drinking.), does not have any emotional illness, does not have any physical disability, does not have any cognitive disorders.

The data collection process was approved by the Department of Research and Technology of Guilan University of Medical Sciences prior to the fieldwork. They were only regarded by code throughout this study. It was emphasized that withdrawal or refusal from the study will not affect the provision of prenatal care. After giving voluntary informed consent to participate in the research, the eligible women were asked to complete the anonymous questionnaire.

Participants were recruited via an advertisement (poster), in the Al-Zahra Hospital. All participants were asked to fill out a two-part questionnaire. The first part of the questionnaire was developed based on the literature review that consisted of a series of factors (age, education level, occupation status, monthly income, current marital status, and relationship length, number of living children, drug abuse, and reproductive history) that can be influenced on domestic violence. In the second part the research instrument assessing domestic violence was the violence against women instrument (henceforth referred to as ‘VAWI’). The World Health Organization's multi-country study developed VAWI based on collaboration with several expert groups, networks, and the original conflict tactics scales.^7^ WHO constructed a questionnaire for the WHO Multi-country Study on Women's Health and Domestic Violence against women. Violence against women instrument is a useful and standardized instrument to assess physical and emotional violence, and controlling behavior by intimate partners or ex-partners, or others.

VAWI consists of behavior-specific items related to psychological (4 items), physical (6 items), and sexual violence (3 items). The physical violence items are further divided into ‘moderate’ (the two first items) and ‘severe’ (the following four items) violence based on the likelihood of physical injury.

For each question, respondents were asked whether they had experienced specific activities during the past year and earlier in life. Participants were considered victims of domestic violence if they provided at least one positive answer to each of the questions in the questionnaire. A number of studies conducted in Iran assessed the validity of this instrument. 22 For the scale level content validity, we use the content validity index (CVI) for clarity and relevancy of each item based on the seven experts' responses and comments. Cronbach's a was used to measure the evaluation of the internal consistency reliability of the research scale. The alpha coefficient was 0.92, and the reliability of this instrument has been reported as 0.88.

Descriptive and inferential statistics were used for data analysis. To identify risk and protective factors were used the chi-square test and logistic regression. The odds ratios (ORs) and 95% confidence intervals (CIs) were calculated by the multiple logistic regression models. A p-value less than 0.05 is trumpeted as statistically significant. Statistical analyses were performed using SPSS software ver. 16.0 (IBM SPSS Statistics for Windows, Version 16.0. Armonk, NY, USA).

## Results

The median age of the women and their spouses was 17.11 ± 1.23, and 28.18 ± 1.07 respectively. The majority (%43.53) of participants had a primary education level and more than 10 % had no formal education. The largest proportion of women (95.29%) reported being unemployed or housewife as their occupation at the time of the survey. Most of the participants (94.9%) reported an unsatisfactory income. More than 63% of participants reported that had childhood exposure to violence (Table 1).

**Table 1:** Sociodemographic characteristics of participants and their spouses

Variables	Mean (SD) or Number (Percent)	
Women's Age (years)	17.11 ± 1.23	
Spouse's Age (years)	28.18 ± 1.07	
Consanguinity marriage	Yes	207(81.18)
	No	48(18.82)
Woman's Education Level	No formal education	27(10.59)
	Primary	111(43.53)
	Secondary	91(35.69)
	Diploma	24(9.41)
	University (Student)	2(0.78)
Spouse's Education Level	No formal education	8(3.14)
	Primary	11(4.31)
	Secondary	42(16.47)
	Diploma	136(53.33)
	University	68(26.67)
Employment status of woman	Employed	12(4.7)
	Unemployed or Housewife	243(95.29)
Employment status of spouse	Employed	196(76.86)
	Unemployed	59(23.13)
Monthly Income	Satisfactory	13(5.1)
	Unsatisfactory	242(94.9)
Home ownership status	Owner	4(1.57)
	Tenant	251(98.43)
Spouse's substance use	No	97 (38.03)
	Cigarettes	127 (49.8)
	Opium	2(0.78)
	Alcohol	26(10.19)
	Other	3(1.18)
Childhood exposure to violence	Yes	161(63.13)
	No	94(36.86)

* SD: standard deviation

The Prevalence of all types of domestic violence is displayed in Table 2. The highest and lowest prevalence of violence among participants were related to emotional violence (23.52%) and physical violence (13.33%), respectively.

**Table 2:** Prevalence of the types of domestic violence among participants

Violence	Mild	Moderate	Severe	Total
	Number (%)			
Physical	19(7.45)	11(4.31)	4(1.56)	34 (13.33)
Emotional	32(12.54)	18(7.06)	10(3.92)	60(23.52)
Sexual	13(5/09)	8(3.13)	2(0.78)	23(9.01)

Table 3 displays a multivariate logistic regression model for the risk and protective factors of domestic violence among pregnant adolescents. After adjusting for possible confounding variables, domestic violence was more likely to be reported for the spouse's substance use (OR 2.41, 95% CI 1.25–4.62), and the spouse's low education (OR 1.41, 95% CI 1.12–3.52). Childhood exposure to violence (OR 1.85, 95% CI 1.46–2.51) and Spouse's unemployment were associated with increased odds of domestic violence (OR 1.14, 95% CI 1.03–1.57). The women's employment was associated with decreased odds of domestic violence (OR 0.70, 95% CI 0.45–0.83).

**Table 3:** Multivariate Logistic Regression Model

Variables	Estimate of parameter	Standardization of parameter estimates	Chi-square	*P* value	AOR (95% CI)
Spouse's substance use	1.146	0.135	7.898	0.002	2.41 (1.25–4.62)
Spouse's low education	0.352	0.118	7.316	0.001	1.41 (1.12–3.52)
Spouse's unemployment	0.033	0.135	7.147	0.001	1.14 (1.03–1.57)
Childhood exposure to violence	0.245	0.127	3.529	0.032	1.85 (1.46–2.51)
Woman's education level	− 0.225	− 0.096	4.382	0.016	0.70 (0.45–0.83)

## Discussion

This study contributes substantially to our understanding of the protective and risk factors of domestic violence among pregnant adolescents.

The majority of participants (23.52%) reported experiencing emotional violence. 13.33 % of participants reported at least one occurrence of physical, and nearly ten percent of participants (9.01%), endorsed at least one item of sexual violence. Monterrosa-Castro et al.^23^ reported physical violence: 6.7 %; emotional violence: 3.7 %; sexual violence: 2.2. %

In a systematic review of the prevalence and risk factors for domestic violence in Ethiopia, 24 the authors reported that more than half of the participants indicated having experienced emotional violence at some point during their lifetime. This finding is consistent with our results indicating a high rate of emotional domestic violence. Recent studies in Iran and other countries have reported similar findings, indicating high rates of emotional violence in these countries.^7^, ^18^, ^25^, ^26^, ^27^ So, emotional violence is a common type of domestic violence, especially in Asian countries, a finding that needs further attention. Although behaviors such as humiliation and verbal abuse by intimate partners may be prevalent in many regions of the world, this type of violence against women may have a more negative and intense impact on mental health because women are more emotionally sensitive than men.^28^

The present study found that the prevalence of physical violence was lesser than emotional violence decreasing. These findings are consistent with other studies. 18, 20, 23 For example, a study conducted in China found that almost one-third of women experienced physical violence.^29^ This finding can be due to physical violence being more outstanding in counseling centers and courts, women hard to talk about the physical violence, and the existing strict laws against physical violence.

The results of various studies, especially those in Iran, show that sexual violence has a lower prevalence compared to other types of violence, which is consistent with the present study. 25, 26, 27 The reason for the low report of sexual violence is the different definitions of sexual violence in Iran compared with European and Western countries. Also, the factors such as cultural barriers in reporting this type of violence have made the available statistics definitely lower than the actual amount, and as a result, this type of violence has a lower reporting. The beliefs and cultural values encourage Iranian women to keep silent and maintain abusive relationships in secret.^25^, ^26^ Domestic violence of any type needs more attention to ensure women's mental health, because, abused women are more likely to experience psychological problems and negative pregnancy experiences. 28

Spouse's substance use, spouse's low-educated, spouse's unemployment, and domestic violence exposure in childhood were factors contributing to the risk of becoming a perpetrator of domestic violence.

The findings of this study showed that the women with spouse's substance abuse were at more risk of experiencing violence from their husbands. Our findings support those of Radcliffe et al^30^ and Choenni^31^ that violence may be more likely where men with Substance abuse.

This study indicates that women with less-educated spouses had more risk of experiencing domestic violence, the result that was consistent with previous studies. 25, 26, 27 Poorly educated Spouses may be unable to solve problems and the more likely to use violence. 32 The lower levels of the socioeconomic status usually may result from lower education levels. Also, women whose Spouses were unemployed were prone to domestic violence.^24^, ^25^, ^26^ Unemployment is a stressful situation for men in and of itself and increases the risk of frustration, hence may heighten the risk of violence. In several previous studies, man's unemployment has been a significant predictor of male-to-female intimate partner violence. Many previous studies have reported that man's unemployment has been a powerful risk factor for intimate partner violence against women.^24^, ^26^, ^27^

Current study findings are in accordance with previous studies that have suggested that domestic violence is more likely to be reported by adolescents who history of violence. There is overwhelming evidence that adolescents who history of violence may lack communication with their families, and receive less parental support.^25^

These results suggest that women's education level may serve as a protective factor against domestic violence. Our findings are similar to other studies that have reported that educated women are less likely to report domestic violence.^33^

Education is a key ingredient in empowering women. Literacy through access to media can contribute to changing and promoting traditional norms, gender roles, and attitudes against violence. Also, increasing education opportunities and female child empowerment can contribute to reductions in gender-based violence and harmful practices, including early marriage.

## Limitations

Although this study identified a 23.52% emotional and 13.33% prevalence of physical violence during pregnancy, we believe this value may be underestimated because more serious forms of violence may hinder from seeking prenatal care, so these women did not come for prenatal care.

Although, the antenatal visits present opportunities represent a unique opportunity to get in contact with the health service for many women, which in turn provides the advantage of creating conditions for the evaluation of violence throughout the entire pregnancy period, but can be considered a phase in which the psychological conditions may influence the responses to the survey.

## Conclusion

In general, most of the participants had experienced domestic violence. Factors associated with experiencing domestic violence include participants' and their partner's education level, experiences of past violence (before marriage), partners' occupation, and monthly income.

Domestic violence or intimate partner violence against women particularly adolescents are now widely recognized as serious human rights abuse. To achieve the Sustainable Development Goals (SDGs), the Elimination of all types of discrimination against women and factors that contribute to the violence against women must be addressed in multiple groups of society. Therefore, the information obtained in this study can be used by policymakers, government officials, decision-makers, and any relevant institution in designing prevention and control strategies to combat domestic violence in different parts of the country.

This study provides numerous ideas for future research. Future research could focus on whether or not victims who did disclose domestic violence felt there were hidden consequences to the disclosure. For example, while there are positive aspects of the disclosure, such as getting help and emotional support, there are negative consequences to disclosure, such as possibly heightening the risk of violence at home.
